# Filling Teeth with Tin

**Published:** 1876-01

**Authors:** H. L. Ambler


### DENTAL REGISTER.

Vol. XXX.] JANUARY, 1876. [No. 1.

### FILLING TEETH WITH TIN.

BY H. L. AMBLER, M. D., D. D. S.

*Read before the Ohio State Dental Society, Dec. 2d, 1875.*

For years tin foil has been admitted to be a good material for filling the temporary teeth, and we take for granted that none deny it at present; but we think that it is just as good or better for filling the permanent teeth under certain conditions. Up to the age of fifteen, and sometimes longer, we find in many cases, that the teeth are quite soft, and in some of these mouths the fluids are so viscid that the preparations of oxy-chloride will not last long, (this condition we have more often found among the Jews and Germans) then we would use tin, even in proximal and labial cavities of incisors, because as the patient grows older the tooth structure generally becomes harder, then the fillings can be removed and good saving operations made with gold.

In correcting cases of irregularity where the teeth are of soft structure, and also decayed more or less; or when you are limited for time and means, you will find tin a valuable material for filling. When there is not good calcification of tooth structure, a condition often found in the six year molars, we would also recommend it. In proximal cavities, when the decay is white and soft, we would separate freely from the palatine or lingual side; and in any cavity in any tooth attacked by this species of decay, we claim that tin will make a saving filling. Professor Watt says: When two metals are so situated in the mouth that the mucous membrane forms a connecting conductor, and the fluids are capable of acting on one of them, galvanic action is established sufficient to decompose any of the binary compounds contained in these fluids; the liberated nitrogen, hydrogen and oxygen form ammonia and then nitric acid; you also have in the mouth, air, moisture and decomposing nitrogenous food to assist in its production. White decay, the most formidable variety known, is produced by nitric acid, and here you find all components of the teeth are acted on and disintegrated, as far as the disease extends.

Dr. S. B. Palmer says: Tin not only arrests decay mechanically, but in frail, chalky structures, acts as an anti-acid element in arresting the electric current set up between the tooth bone and the filling material.

Some operators have advocated filling teeth with a mixture of tin and gold in the same cavity, allowing both metals to be exposed to the fluids of the mouth; they claim that fillings can be made quicker, that they are not as subject to thermal changes, and that they can be inserted nearer the pulp without danger, than when gold alone is used; all of which three claims are entirely met by only using the tin. One of these writers says: That the mixture will preserve the tooth quite as well as an entire gold filling, making no conditions or restrictions as to tooth substance or location of cavity.

Thus far we can not see any advantage which this mixture has over the clean pure tin or gold, we have lately seen a case of a superior bicuspid filled in posterior proximal and masticating surface; about one year after filling, the tooth split between the cusps, we removed the palatine cusp including about one third of that side of the root, here we found that calcification of the pulp had ensued, owing to the close proximity of the tin filling. It has been known for years that tin would be tolerated in large cavities very near the pulp without causing any trouble. We would not recommend it for filling root canals, for should there be any leakage, the tin will oxydize, and you may have infiltration into the crown causing discoloration; this is the more noticeable when the crown is filled with gold. In many mouths it does not oxydize, but preserves a clean grayish appearance, but should the surface oxydize, we know of no bad results therefrom. Where the fillings are subject to much attrition they wear away soon or late, but this not a great drawback, for they may generally be easily and quickly replaced, and from the concave shape which they assume, the patient is liable to know when a large amount has been worn away, that portion against the walls and around the margins being the last removed, thus preventing further decay as long as there is any reasonable amount of tin left, neither does it expand or contract (like some base mixtures) enough to produce leakage along the margins. On account of its softness and ductility, tin may be driven into or on to the dentinal tubuli, where they are a little rough, so as to completely close them to outside moisture, and you may often see in burnishing a filling by hand force, when it is perfectly dry, that the tin has taken such a hold in or on the dentine, that to remove it requires a sharp cutting instrument. For filling by hand pressure, use instruments with square ends and sides, with shallow serrations. For filling with the mallet, use instruments with medium serrations, and a steady medium blow with a four or six ounce mallet. Any instrument turned at any angle which will best reach the cavity, is admissible, but with the above modifications.

In all operations you must have absolute dryness, generally by the use of Barnum's rubber dam.

If the cavity is of a good general retaining shape, all very well, but if it is not, then cut some opposing angles or pits, making them somewhat larger than for filling the same cavity with gold. The extra tough foil No. 18, manufactured by S. S. White, retains a bright clean surface and does not lose its good working qualities, even after much exposure to the atmosphere, and when well condensed into a filling either by hand or mallet force, it stays up to the walls of a tooth and actually makes a tight filling, with a surface which will stand the wear from three to ten years, depending upon the size and location of the filling. Strips of this foil from two to four thicknesses can be welded together by mallet or hand, and will cohere to the same extent or better than semi-cohesive gold foil, thus if there is only one or two walls of a cavity left, you can bring out your filling to any practical or desired contour.

Fold up the tin into strips of different widths and lengths, according to the size of the cavity, but for large crown or proximal cavities, these strips may be folded into mats or rolled into cylinders, enabling you to make a quicker operation, but much more force is necessary in condensing; whereas, with the strips you can fill any kind of a frail tooth, and at the same time have the filling all one solid mass. After the cavity is full, go over the tin thoroughly with mallet or hand force; cut down crown fillings with the engine bur or corundum point, and proximals with sharp cutting instruments, and strips of sand paper, after partially cutting down, giving the filling another condensing, then a final trimming and burnishing, by this means you secure a hard mouth surface and complete the operation.

